# Refugees, water balance, and water stress: Lessons learned from Lebanon

**DOI:** 10.1007/s13280-019-01272-0

**Published:** 2019-11-02

**Authors:** Hadi Jaafar, Farah Ahmad, Lauren Holtmeier, Caroline King-Okumu

**Affiliations:** 1 Department of Agriculture, American University of Beirut, Beirut, Lebanon; 2 The Bush School of Government & Public Service, Texas A&M University, College Station, TX USA; 3 University of Southampton, UK, University Road, Southampton, SO17 1BJ UK

**Keywords:** Conflicts, GIS analysis, Lebanon, Middle East, Migration, Water security

## Abstract

The Syrian crisis caused a massive influx of displaced people into neighboring countries, with Lebanon hosting the highest per capita number of refugees (3:10). Water remains the most critical natural resource that influences the resilience of host and refugee communities. We provide a new GIS-based updated water balance and water scarcity analysis at the national and the watershed level in Lebanon by comparing current conditions to no-refugee levels. Results show a small (6%) increase in water stress in an average water year at the national level that masks hot spots of water scarcity at the local geography. While domestic water use increased by 20%, we find that refugees’ water use is only 10% of agricultural water use in summer. We also show that interventions to rehabilitate the water networks can reduce water stress to better than pre-conflict levels (3% less stress).

**Electronic supplementary material:**

The online version of this article (10.1007/s13280-019-01272-0) contains supplementary material, which is available to authorized users.

## Introduction

Millions of refugees are migrating throughout the world escaping threats caused by violence, conflicts, or other circumstances (UNHCR 2011). Developing countries host the vast majority (89%) of the world’s refugees. The countries hosting the most refugees worldwide are Turkey, Lebanon, and Jordan (hosting 27% of total world refugees), Pakistan and Iran (hosting 16%), and Ethiopia and Kenya (hosting 7%) (World Bank 2016b). Before the Syrian conflict, Lebanon was the 28 most water-stressed country in the world (Gassert et al. 2013). The crisis had unexpectedly triggered waves of refugees to neighboring countries. With more than 1.5 million Syrian refugees, Lebanon now, by far, hosts the largest number of refugees per capita worldwide. Since then, water stress has increased, but it remains to be quantified at the national and, more importantly, the sub-national level. There are reports of declining groundwater levels in several aquifers in Lebanon as well as a decline in spring flows that has been linked to the refugee crisis (UNDP 2014b). However, water stress existed in the country even before the Syrian crisis, mainly due to population increase coupled with network deterioration and water governance issues across the significant water institutions and authorities within the country. While many studies have analyzed the demographic, social and political complications of the refugee crisis in Lebanon—see for example: (Refaat and Mohanna 2013; Berti 2015) on Syrian refugees, and (Roberts 2010) on Palestinian refugees—none, to the authors’ knowledge, have quantified the impact of the crisis on water stress in the country by comparing the situation before and after the refugee crisis at an appropriate spatial scale. The primary objectives of this research are: (1) to provide an updated water balance for the country, (2) to spatially quantify water stress in Lebanon following the Syrian refugee crisis, (3) to compare current conditions to a scenario where reasonable network efficiency improvements are made, and (4) to provide recommendations for policy directives and improvements.

Findings show that while national and even governorate-level analysis of water use mask local and basin-level patterns of water stress and abundance, water savings measures, if implemented, can reduce water stress in the country to before-Syrian-crisis levels. The methodology used herein is based on analyzing spatial variation of domestic and industrial water use, agricultural water consumption (using an established energy balance model), and population data in Lebanon using geographic information systems (GIS) in the presumed absence of the Syrian crisis (scenario 1). To estimate the refugee impact, the analysis is re-run based on the existing conditions with more than 1.5 million refugees in the country (scenario 2), and re-run once again assuming a degree of improvement in network efficiency (scenario 3). The analysis shows the location of increased water use, the quantity of that use, and the benefit of improving network efficiency in urban areas. This contribution is essential to both the scientific and the development community, because it highlights hot spots of severe water stress in the country, and because it provides a basis for quantifying refugees’ impacts in other similar situations. We conclude that the refugee crisis has created and continues to sustain extreme hot spots of water stress in urban areas that need immediate attention. However, we also observe that the refugee water use is still a small percentage of the total water use, especially when compared to agricultural water consumption in the country. Moreover, the geographic distribution of refugees creates different degrees of water stress in various parts of Lebanon, partly due to the uneven distribution of both water resources and refugees.

## Background

### Impact of refugees’ crisis on the water in host countries

While there are a plethora of studies relating to the Syrian refugee crisis impacts in the region, many of these studies focus on socio-economic components rather than bio-physical ones—see for example (El-Khatib et al. 2013; Achiume 2015; Wall et al. 2017). Spatially evaluating the impacts of refugees on the hosts’ land use and water resources remains a challenge (Müller et al. 2016). Many studies from different parts of the world have shown how refugee crises have imposed stresses on water resources in host countries (Black 1994; Hoerz 1995; Jacobsen 1997). In Jordan, for example, increased groundwater pumping due to the Syrian crisis caused declines in the water table in one of the largest and highly populated camps (Abu-Allaban et al. 2015; UNDP 2015).

The sudden increase in the demand for water in refugee camps and settlements may create water shortages for both the refugees and the host communities that need to be quantified for adopting the most appropriate resilient measures. The impact of refugee crises have been studied previously in several countries such as Tanzania (Paskett 1998), and Sudan (World Bank 2010), and the Iraqi refugee influx to Jordan in 2003 (Sassoon 2008).

Very often, the impact of the crisis on water use is indirect. Conflicts have induced a change in land and water use and management of both war-affected and host countries. For example, the Syrian crisis led to a decrease in irrigated agriculture in Southern Syria, associated with changes in the way Syrians managed their reservoirs, which caused an increase in the flow of downstream Yarmouk river to Jordan (Müller et al. 2016). A remote-sensing based study conducted on the Syrian portion of the Orontes basin showed a severe drop in irrigated agricultural production (Jaafar et al. 2015), indicating a lower usage of both surface and groundwater in the Syrian parts of the basin in contrast to the Lebanese part were water use had been reported to increase (Jaafar et al. 2016; King and Jaafar 2015).

### Lebanon’s political and administrative structure

**Fig. 1:**
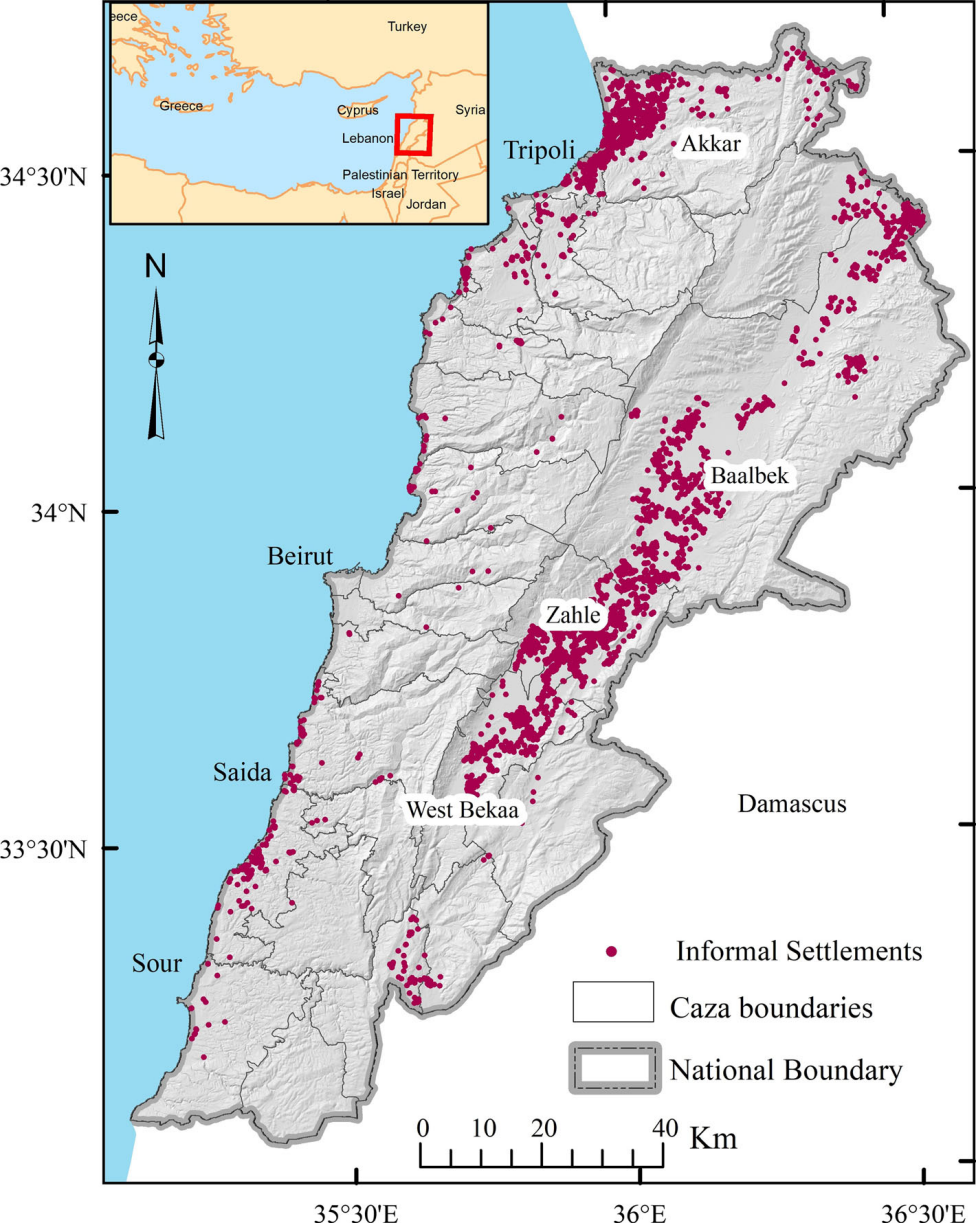
Geographic map of Lebanon showing the location of informal refugee settlements in 2016 within the various administrative divisions of Lebanon (source for locations of informal settlements: IAMP 2016)

Lebanon is a small country (10 425 km^2^) that lies on the Eastern Mediterranean with 82% of its borders shared with Syria (Fig. 1). Before the Syrian crisis (2011), the population in Lebanon was estimated at 4.6 million, most of which are considered to be urban (88%) (World Bank 2017). The World Bank estimates the population as of 2016 to be 6.01 million. Lebanon has eight governorates (Beirut, North, Akkar, South, Nabatiye, Beqaa, Baalbeck-Hermel, and Mount Lebanon). Each of these eight governorates is further composed of several administrative units called “Kada,” and each Kada consists of several municipalities. The total number of Kadas in Lebanon is 25, and there are 1 561 municipalities within the country with an average area of 6.7 km^2^ per municipality. The population of Lebanon in 1932 was 875 000. Following the end of the civil war in 1990, the population has grown, reaching approximately 4 million Lebanese in 2002 (Central Administration of Statistics 2017). Since the beginning of the Syrian conflict in 2011, there has been an unexpected increase in the number of refugees in Lebanon. In his address to the United Nations and world leaders, the Lebanese president Michel Aoun in 2018 stated that Lebanon “can no longer cope” with the refugee crisis, stating that the current density of Syrian refugees in Lebanon is 153 per km^2^ (an equivalent of 1.6 million) (Dakroub 2017). Even before the crisis, Lebanon already faced water stress. Post-civil war expansion in population and lack of adequate water policies had transformed Lebanon from a water-secure country (~ 2 500 m^3^/capita in the 1960s) to a water-deficit country (less than 1 000 m^3^/capita) just before the Syrian conflict (FAOSTAT 2017). The primary water sources used by refugees in Lebanon are the public water network which is mainly fed by springs and groundwater (UNDP 2014a), especially within the Litani Basin where a large portion of the refugee population is located (Jaafar et al. 2016).

## Materials and methods

### Population data

To evaluate the impact of the refugees, a no-Syrian refugee population scenario for 2016 is developed as follows. Using Lebanese government statistics for years 2002–2016 derived from the Ministry of Interior, General Directorate of Civil Status and published by the Central Administration Statistics (CAS) (Central Administration of Statistics 2017), the net increase in population in each governorate is calculated by subtracting the total births from the total deaths registered in each governorate of the country. While a net migration rate for 2002–2010 averaged 20 per thousand was reported by the UN (Desa 2014), the World Bank reports a negative net migration of − 0.6 per 1 000 for the 5 years before the crisis in 2011 (World Bank, 2016a). No internal migration data in Lebanon are collected (Bell et al. 2015). The population expansion rate per governorate was used to calculate the governorate’s average population growth rate (annual rate of natural increase which is the crude birth rate minus the crude death rate). The expansion rate is then assumed to apply at each municipality within that governorate. This assumption is the best estimate given (1) the lack of any other higher resolution data (i.e. net increase in population at the municipal level), and (2) the small scale of the Lebanese governorates. The resulting population figure for Lebanon in 2016 (~ 5 million) is slightly higher than the World Bank’s estimate (4.6 Million). Population growth rates for Lebanon between 2002 and 2016 ranged between a minimum of 1.26% following the Israeli war in 2006 and a maximum of 1.63% in 2014, averaging at 1.46% per year (Table 1). Growth rates for governorates were constant for South Lebanon and Nabatiye, decreasing for Beirut, and increasing for other governorates. The population of Palestinian refugees already existing in Lebanon as of 2016 was added to the Lebanese population dataset. This population scenario is the baseline for calculating the “normal” urban water stress situation in the country. Based on the urban areas from a land-use map of Lebanon, a population grid (30 m resolution) was created by equally distributing the population for the municipality over the corresponding urban areas within that municipality. This distribution is the most feasible at the moment given the lack of data on the variability in population density within urban settlements. The resulting population grid was used later in the distribution of domestic water use for the Lebanese population as well as Palestinians and Syrians refugees living informal settlements.

**Table 1:** Population net births for years 2002–2016 for Lebanese governorates (Central Administration of Statistics 2017)

Calculated net births	Beirut	Mount Lebanon	North Lebanon	South Lebanon	Nabatieh	Bekaa	Total for Lebanon
2002	6 367	9 776	18 766	8 726	10 940	4 536	59 111
2003	5 686	9 408	17 145	8 222	9 884	4 170	54 515
2004	5 957	9 259	18 772	8 172	9 826	4 140	56 126
2005	5 577	9 409	18 044	8 954	10 365	3 612	55 961
2006	5 745	8 522	17 977	7 898	9 010	4 851	54 003
2007	5 136	8 228	16 200	8 202	9 703	12 335	59 804
2008	5 725	9 025	16 770	8 546	10 559	13 150	63 775
2010	5 931	9 678	20 763	9 182	13 007	16 215	74 776
2011	6 155	9 568	22 967	9 217	9 251	16 662	73 820
2012	5 506	8 873	21 643	8 714	10 495	15 256	70 487
2013	5 351	10 863	20 473	9 191	10 164	15 191	71 233
2014	5 013	12 701	22 953	10 472	10 015	16 482	77 636
2015	4 589	12 717	21 781	9 180	9 349	15 049	72 665
2016	4 462	10 710	21 741	8 639	9 950	16 449	71 951
Population in 2002	403 338	1 511 686	812 175	477 515	279 239	544 208	4 028 161
Population at end of 2016	480 538	1 650 423	1 088 170	600 830	421 757	702 306	4 944 024
Average growth rate (%)	1.24	0.63	2.07	1.63	2.90	1.80	1.61

### Syrian refugee population in Lebanon

To calculate the total population in Lebanon after the Syrian crisis, the spatial dataset of the Syrian refugee population is summed with the Lebanese population spatial dataset. The Syrian refugee population constitutes of two major classes: those living informal settlements and those living within informal settlements. To determine the population count for Syrian refugees at the end of 2016, the United Nations High Commissioner for Refugees (UNHCR) statistics of registered Syrian refugees residing in Lebanon (IAMP 2016) is adopted. The UNHCR states that the actual number of refugees in Lebanon is much higher than the number of refugees officially registered due to instructions by Lebanese government (preventing registration of refugees, regulations on ceasing the registration process by UN agencies). In 2016, UNHCR reported about 1.24 million Syrian refugees in the country, which is the figure adopted in this study (IAMP 2016). The UNHCR estimated that 20% of the Syrian refugees in Lebanon live in what is referred to as informal settlements in 2016. These include collective shelters, tents, schools, and other structures that are characterized by poor living conditions. The rest of the refugees live among Lebanese hosts. Figure 1 shows the geographic distribution of refugees in informal settlements. The UNHCR publishes the refugee distribution by municipality and those living in informal settlements. The difference between the two counts is the number of registered refugees living in rented or owned apartments and houses. The registered refugees are considered to be distributed equally over the urban area within each municipality.

### Grid for informal settlements

Informal settlements locations and the number of tents in each settlement are published by the UNHCR (IAMP 2016). The areas of settlements vary from one location to another depending on the site location and the population density in each settlement. To delineate the area of each informal settlement, we multiplied the average area of one tent by the number of tents in each informal settlement. A sample of camps in different regions of Lebanon was chosen where the area of camps was calculated using the high-resolution imagery of Google Earth. This area was divided by the number of tents provided by the UNHCR to calculate the average area around the tent (~ 100 m^2^). A minimum area of a settlement was set to a 30 m-resolution pixel. Settlement locations were buffered to obtain polygons matching the calculated area (proportionally to 900 m^2^). The number of individuals in each settlement was then assigned to the corresponding polygon. A 30 m × 30 m grid for the informal settlements was obtained from the derived polygons that were distributed on various municipalities.

The water used for refugees living in informal settlements and for those living in formal settlements as of 2016 was calculated separately, given the unequal water demand and water-use potential within the two categories. The water use of registered refugees was summed with the Lebanese water-use grid in urban areas, while the water use of refugees in the informal settlement was distributed according to settlements location in each municipality.

### The Palestinian refugee population in Lebanon

Palestinian refugees have inhabited the country since 1948. These refugees live mostly in camps that were built for temporary conditions. Other Palestinians live outside camps which are referred to by the UN Relief and Works Agency (UNRWA) as “gatherings.” UNHCR keeps a record of Palestinian refugees who have fled their camps in Syria along with their locations. UNHCR reports the number of Palestinians living in camps and gatherings along with the respective locations based on UNRWA figures and their registers. They are considered to be living in gatherings and not in informal settlements. The water use of Palestinian refugees was added to the Lebanese water-use grid in urban areas. Those Palestinians mainly originate from the Yarmouk camp in the Damascus suburbs. About 40 000 Palestinians have moved from Syria to Lebanon due to the crisis. Although the new Palestinian refugees comprise no more than 3% of the total Syrian refugees in Lebanon, their water use was accounted for because they may exert water stress in localized hot spots. We adopted UNRWA’s official estimate of the current number of Palestinians residing in camps and so-called “gatherings.”

### Determining the current population grids

Population counts in informal settlements per municipality borders were added to the respective Syrian and Palestinian refugees count due to the crisis. The result was two population datasets: one reflecting a no-conflict scenario, Lebanese + Palestinians, and another representing the current status, Lebanese + Palestinians + Syrian refugees + Palestinian refugees living in Syrian camps who have fled Syria to Lebanon. Nationals from other countries residing in Lebanon were not accounted for because they are believed to account for only a small number of constituents (Central Administration of Statistics 2017).

### Domestic water use

The official Lebanese estimate of the per capita daily water use in Lebanon is 180 l/day. This figure is adopted by the Ministry of Energy and Water (MoEW) and is also adopted in the literature (Bou-Zeid and El-Fadel 2002; Korfali and Jurdi 2010; Safi et al. 2018). However, this estimate neglects the spatial component of actual water use, which is also linked to geography, climate, and standard of living. For example, in warmer/more humid climates, water use may be higher, so does the use in developed urban zones as compared to rural regions. Domestic water uses were therefore determined according to population and per capita use statistics as follows. The central administration of statistics (CAS) published the tap water consumption and for each governorate based on data provided by the water authorities in Lebanon (Statistics 2008). According to this data, North Lebanon governorate has the highest water use (187 l/day/capita), followed by Beirut and Mount Lebanon (185 l/day/capita), South Lebanon (175 l/day/capita), and the Bekaa (127.0 l/day/capita). In this study, we adopted these figures for the respective governorates. A network efficiency of 50% was assumed for scenarios 1 and 2 (i.e., with and without refugees) (MoEW 2010). For scenario 3, we simulated the water stress assuming a hypothetical 30% reduction in losses (i.e., 65% network efficiency).

The same per-capita water-use figure for the Palestinian refugees and Syrians living among the Lebanese population was adopted as the same network serves them, and they share the same cultural habits. For calculating domestic use for informal settlements, we assumed that the water used for informal settlements is 50% of that of the adopted figure in the corresponding governorate. This assumption is closest to reality based on the following. Initially, we used UNHCR data (IAMP 2016) to calculate the maximum capacity of water tanks in those informal settlements. The total capacity was divided by the residents of the informal settlement to derive a rough estimate of daily water use, assuming the tanks are being filled once per day. The average daily water use given this assumption is 120 l/capita/day. However, water distribution across the country is not uniform. Knowing that sources of water for these settlements vary (sometimes water is delivered by trucks, in other situations it is supplied by the existing network, and sometimes a new borehole is drilled to supply water), it is usually not customary to have these tanks filled once per day. The most common filling pattern is 2–3 times per week. There is also a lack of adequate water appliances in these tents (for example, washing machines, bathtubs, and others).

The water use for existing Palestinian refugees was combined with that of the existing Lebanese population in the corresponding municipality (no-refugee scenario-1). That is, the no-Syrian refugee scenario was based on water use by both the Lebanese and the Palestinian refugees because the latter were considered permanent residents in the country. The increase in water use due to the influx of Palestinian refugees from Syria was accounted for by adding their water use to that of the Syrian refugees (scenario 2). Annual water uses were estimated spatially in units of depth (mm), and volume (m^3^). The volume of water was divided by the area of the urban zones in the respective municipality to generate a use grid in mm.

### Industrial water use

Industrial use, which is assumed not to be affected by the refugee crisis, is included in the analysis herein because it is a significant component of urban water use. Currently, there is no water accounting for industrial water use in Lebanon. The major industries in the country are chemical, leather, food including wine, aluminum, paper, plastic, ceramics, cement, rock and sand quarries, and other materials. No metering exists in any industry, and it is nearly impossible to account for industrial use. Therefore, we adopted the official MOEW estimate, which assumes that the industrial use is 30% of that of the domestic (MoEW 2010), with the same network efficiency. The Ministry of Industry recently published the number of industrial firms among Lebanese Kadas (https://www.lebanon-industry.com/statistic.pdf). In this study, the geographic distribution of the industries was extracted from the land use map of Lebanon (2013) provided by the Lebanese Center for Scientific Research (L-CNRS).

Another 30 m × 30 m grid was constructed for industrial distribution. An average water use per industry is calculated as the ratio of the total industrial use to the total number of industries in Lebanon. This ratio was multiplied by the number of industries in each Kada to calculate the industrial water use at the Kada level. Then the use was distributed in proportion to the industrial areas (as per the land use map) within each municipality. The result is a municipality-level industrial water-use grid. This approach is the least subjective approach given the lack of data on the distribution of the various industries per type and their water usage statistics.

### Agricultural water use

**Fig. 2:**
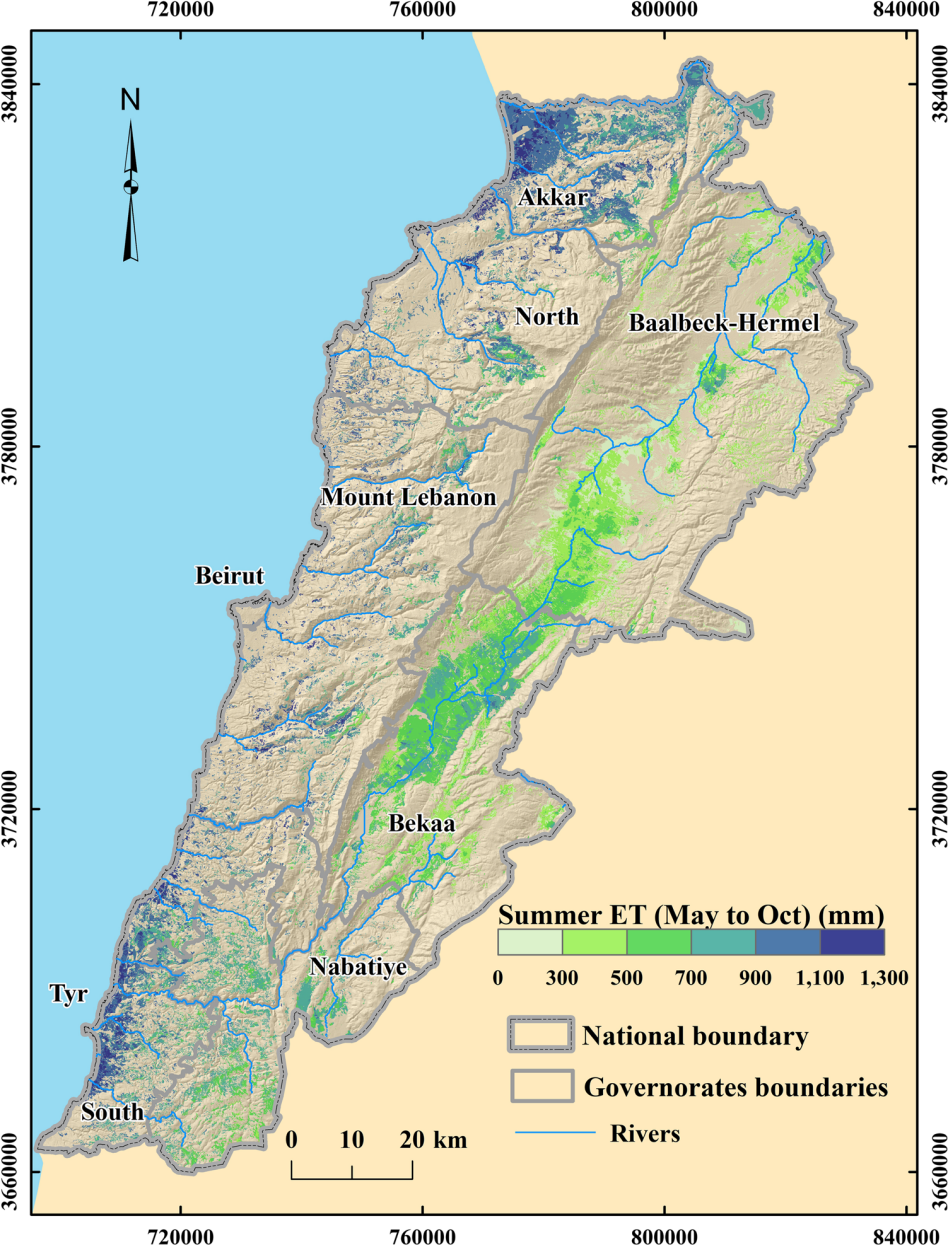
Distribution and magnitude of irrigation use in Lebanon summed over the irrigation season of May to October and averaged over 2010–2016

Agricultural water use in Lebanon still lacks accurate accounting. To calculate agricultural use, we produced, for the first time, a field-scale actual evapotranspiration (ET) grid (i.e. agricultural water use) at the 30-m resolution (Summer ET) for May–October, which is the main irrigation season for Lebanon (Fig. 2). Agricultural ET during the wet season (November–April), is assumed to be met by green water, i.e. rainfall stored in the root zone of crops and available for biomass production (Falkenmark and Rockström 2006). A new version (python-coded) of the Surface Energy Balance Algorithm for Land (pySEBAL) was implemented on Landsat imagery (30 m spatial resolution) in this study. SEBAL is a well-established and validated single-source energy balance model that has been widely used to calculate field-scale ET (Bastiaanssen et al. 1998, 2005). We prefer to use Landsat satellite imagery and SEBAL to estimate agricultural water use because finer resolution imagery is more suitable to estimate ET in heterogenous croplands that dominate the Lebanese Landscape than MODIS (McCabe and Wood 2006). In brief, SEBAL calculates ET as the residual of the energy balance equation (ET = *R*_n_ − *G* − *H*), where *R*_n_ is the net radiation, *G* is the soil heat flux, and *H* is the sensible heat flux. The energy balance was spatially calculated using a combination of field weather data and remotely sensed data. SEBAL utilizes remotely sensed Land surface temperature (from thermal bands of satellite imagery), and the normalized difference vegetation index (from reflectance in the red and near-infrared bands of the satellite, along with other variables such as albedo, incident solar radiation, temperature, wind speed, and relative humidity). To generate 30-m-resolution ET grids for Lebanon, we used all cloud-free Landsat Imagery (Landsat 5, filled Landsat 7, and Landsat 8 imagery) for 2010–2016. We mosaicked the Landsat scenes (two Landsat scenes cover Lebanon), clipped to the country boundary, filled the gaps in Landsat-7, run pySEBAL, and then averaged the ET derived from 97 satellite overpasses over the summer period of May–October to calculate the average summer ET “map.” We gap-filled the L7 images using the “focal” function from the “raster” package in R (Hijmans 2016). The function estimates the values for the neighborhood of focal cells using a 3 × 3 moving window. We filled the thermal, red, near-infrared, and surface reflectance shortwave bands by averaging the moving window, while the pixel’s quality-assurance bands were filled by the maximum of the neighborhood of focal cells (worst case scenario). For a detailed description of the methodology and the assumptions for calculating ET for Lebanon, the reader is referred to Jaafar and Ahmad (2019). We do not account in the analysis for the losses in the irrigation transmission, distribution, or on-farm systems, because, at the basin scale, only ET is the actual loss from the system.

### Water availability estimates

Many approaches consider only surface water to be the primary sustainable source for communities (Vörösmarty et al. 2000). However, we opt to determine renewable water as the difference between precipitation and natural evapotranspiration, which constitutes the renewable surface flows and the renewable groundwater recharge. Gridded annual precipitation data were obtained from a remotely sensed gage-calibrated precipitation product CHIRPS v2.0 (Funk et al. 2015). We averaged the data for 2010–2016. Average rainfall over Lebanon for this period was 703 mm/yr., compared to the long-term average of 705 mm/yr for 1984–2016 (Fig. S1). CHIRPS grids were re-sampled to 30 m using a kriging model in GIS where the method used was “universal kriging.” Universal kriging is a geostatistical technique for spatial interpolation with minimum and known variance based on the theory of regionalized variables. The method is based on doing the regression with the spatial coordinates as the explanatory variables, and it assumes the errors to be autocorrelated rather than independent (Oliver and Webster 1990). Natural evapotranspiration was calculated as an average for the same period (2010–2016) using NASA’s MODIS 8-day ET product (MOD16A2) (Running et al. 2017). The natural ET was calculated as the sum of the annual MODIS ET of natural lands and the winter MODIS ET of agricultural lands (because winter ET is primarily supplied by rainfall, we consider this consumption as a loss from the supply rather than as a demand). We use MODIS rather than SEBAL for winter (November–April) because we found that the latter overestimates winter ET (Fig. S2). Natural lands include forests, shrubs, grasslands, bare soil, water bodies, and riparian vegetation (i.e. all lands that are not occupied with urban and industrial buildings and that are not agricultural).

### Use grids and water stress

To determine water stress, three water-use scenarios were simulated. National water use for the first scenario (scenario 1) is the summation of the domestic use (assuming no Syrian crisis), the industrial use, and the agricultural use. Scenario 2 is the same as scenario 1 but with factoring in the domestic use for the Syrian and Palestinian refugees displaced into Lebanon. Scenario 2 is the closest to current conditions. Scenario 3 is the same as scenario 2 except that network efficiency was assumed to increase from 50 to 65% following proposed rehabilitation.

### Water stress

Annual water stress grids for each scenario were calculated by dividing the annual use grids (domestic use (*D*), industrial use (*I*), summer agricultural water use (ET_Ags_)) by the water availability grid also referred to as renewable water (which is equal to the annual precipitation (*P*) minus the annual natural evapotranspiration (ET_N_) and ET from agricultural lands in winter ET_Agw_) (Eq. 1).

1 $$ {\text{Water}}\,{\text{stress}}\,({\text{FWS}}) = ({D} + {I} + {\text{ET}}_{\text{Ags}}) /({P} - {\text{ET}}_{\text{N}} - {\text{ET}}_{\text{Agw}}) $$

The zonal mean of water stress was then determined for each watershed/hydrologic zone. The result is a unit-less stress indicator grid that shows the relative water use (water usage divided by renewable water). The no-refugee scenario is compared with the current status scenario to highlight regions where water stress is the greatest and also regions with the highest water use. Based on the stress grid, the mean stress per zone was calculated by populating municipalities within the watershed or zone (Eq. 2).

2 $$ {\text{Mean}}\,{\text{FWS}} = \sum {\text{FWS}}\,{\text{per}}\,{\text{pixel}}/{\text{number}}\,{\text{of}}\,{\text{pixels}}\,{\text{per}}\,{\text{zone}} . $$

## Results and discussion

### Water balance updates

**Fig. 3:**
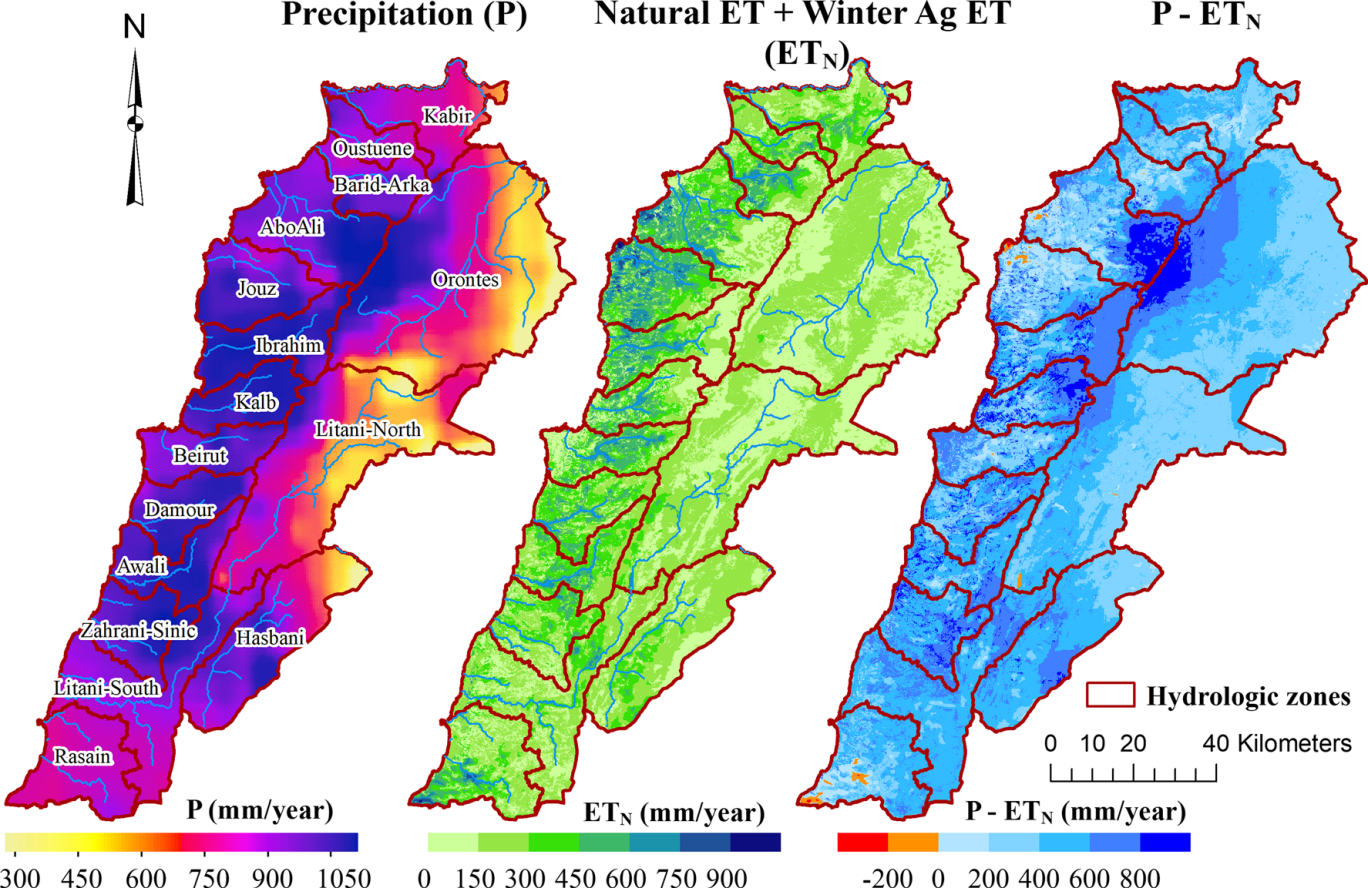
Renewable water in Lebanon: remotely sensed-biased corrected gridded precipitation (CHIRPS), MODIS annual evapotranspiration over natural territories plus winter evapotranspiration from agricultural lands, and the net annual difference between the two (average of 2010–2016)

Summer agricultural water use (ET) is shown in Fig. 2. Our novel analysis shows that irrigated agriculture in Lebanon consumes an average of 1 600 million cubic meters (mcm) between May and October. Due to transmission, distribution, and on-farm field losses, diversions to agriculture are always higher than the actual ET. The Litani, Orontes, the northern and the southernmost watersheds consume two-thirds of the total ET. These regions are where most of the Lebanese agriculture is concentrated, and water diversions to agriculture are in conflict with diversions for domestic and hydropower uses (Jaafar 2014). Precipitation provides 7 200 mcm, natural landscape, and winter agriculture mostly (rainfed) consume 2 380 mcm. Figure 3 shows the precipitation grids, the natural ET grid (including agricultural use in winter, and the difference between the two at a 30 m-pixel level). Variation of renewable water in Lebanon (2010–2016) is shown in Fig. S3. The net balance is positive for Lebanon.

### Impact of the crisis on domestic water use

**Fig. 4:**
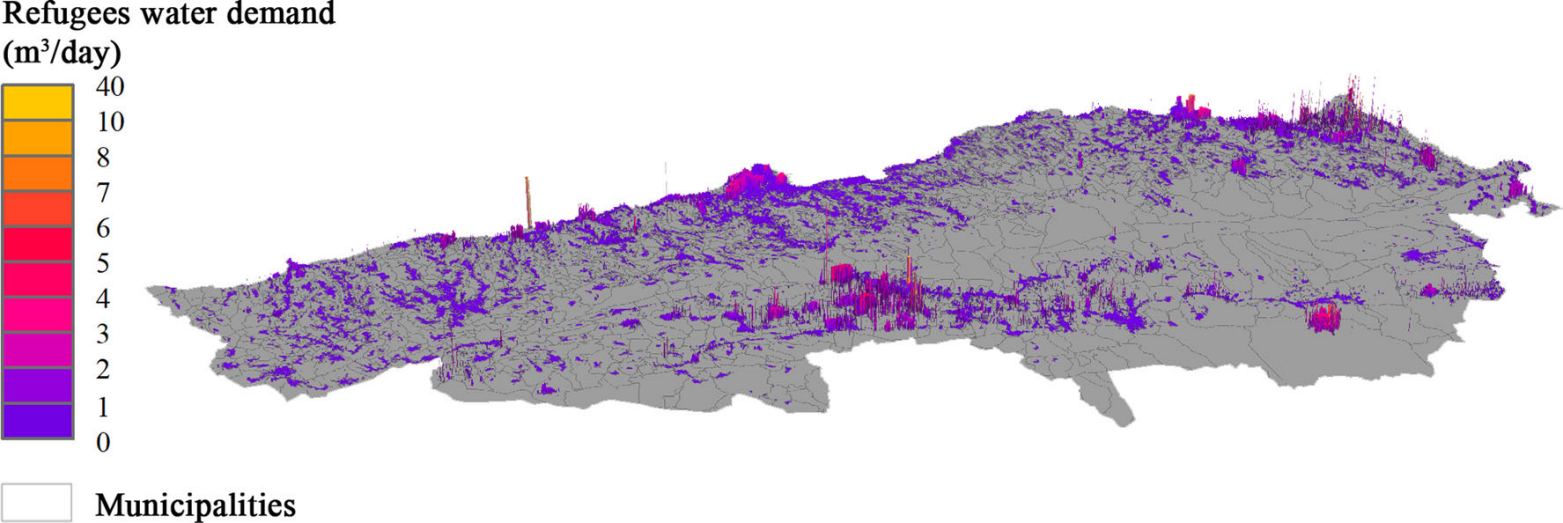
Spatial distribution of refugees’ water use in Lebanon

**Fig. 5:**
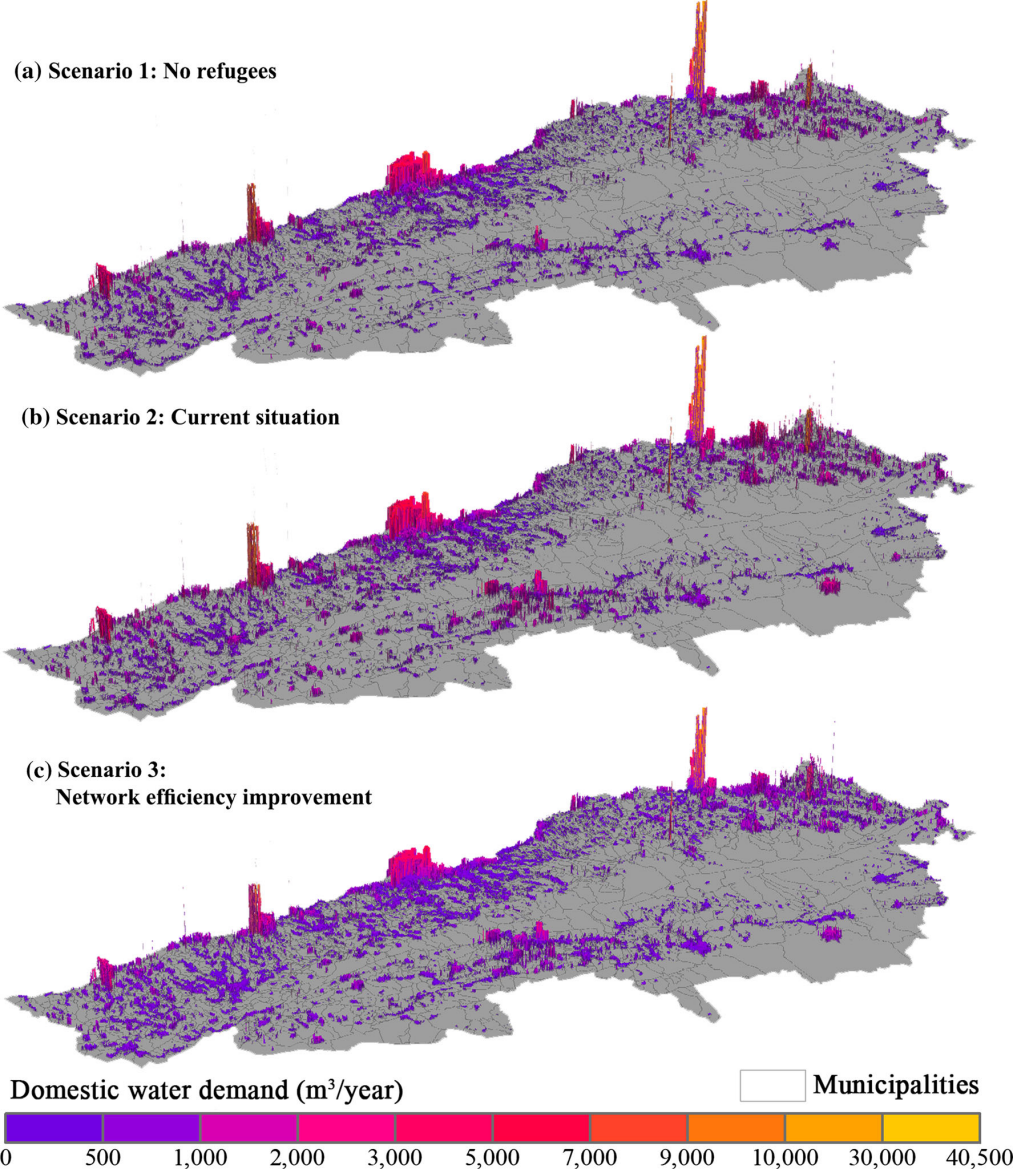
Domestic water-use distribution in urban areas of Lebanon for three scenarios: (1) no refugee situation, (2) with refugees (i.e., current conditions), and (3) with refugees if network losses are reduced from 50 to 35%

**Fig. 6:**
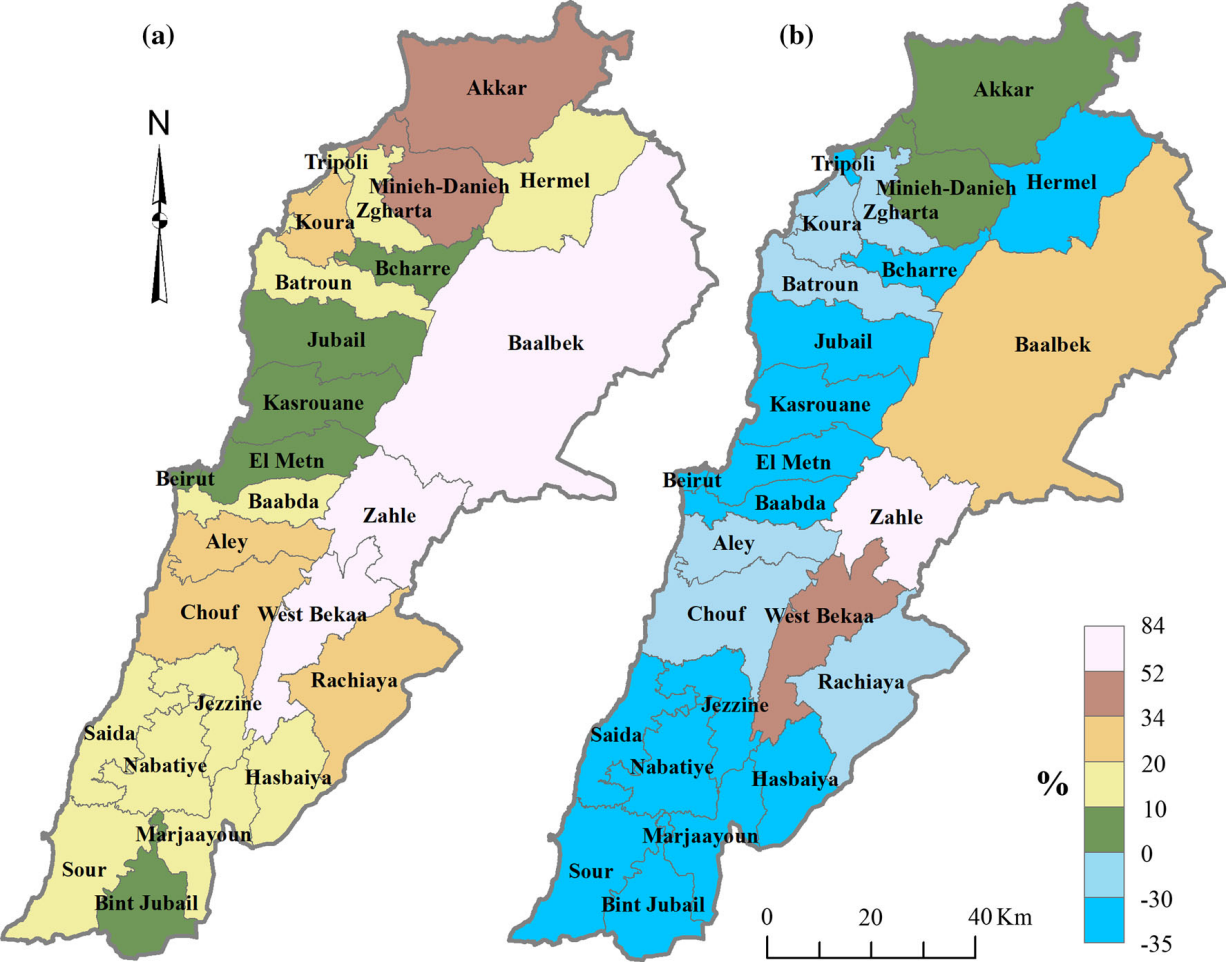
Kada-level Percentage change in domestic uses compared to a no-refugee scenario: **a** current situation as of 2016, and **b** simulated percent change assuming an improvement in network efficiencies. Labels indicate Kadas. Rehabilitation will reduce uses to pre-conflict levels except in Zahle, West Bekaa, Baalbek, Akkar, and Minieh-Danieh

Results indicate a 20% increase in domestic water use in Lebanon due to the refugee crisis (from 676 to 826 mcm/year). The primary determinant of changing levels in domestic water-use results from the population of Syrian refugees living in suburban areas close to cities and areas where land is generally more available (Fig. 4). The most impacted governorates by the increase in domestic use are Bekaa (+ 89%), Baalbeck–Hermel (+ 59%), Akkar (+ 39%), and North Lebanon (+ 20% increase) (Fig. 5). Within the governorates, the most impacted Kadas are Zahle (+ 99%), West Bekaa (+ 91%), and Baalbek (+ 66%). A 30% improvement in network efficiency (i.e. 65% of network efficiency) will reduce uses to pre-conflict levels in all but five Kadas: Zahle (+ 53%), West Bekaa (+ 47%), Baalbek (+ 27%), Akkar (+ 7%), and Minieh-Danieh (+ 4%) (Fig. 6).

**Fig. 7:**
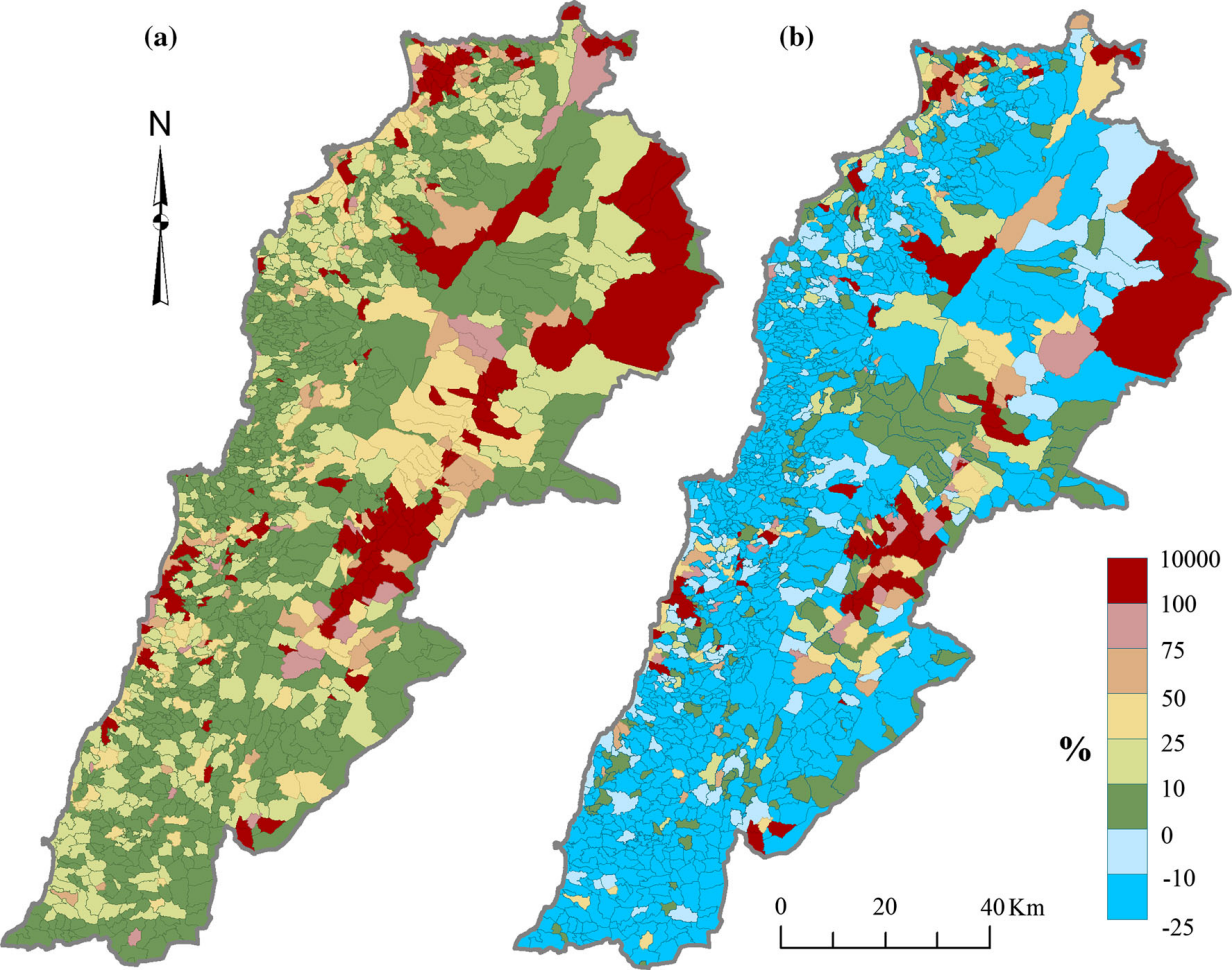
Municipal-level percentage change in domestic uses compared to a no-refugee scenario: **a** current situation as of 2016, and **b** simulated percent change assuming an improvement in network efficiencies and reductions in domestic water use (efficiency increase from 50 to 65%)

At the municipal level (Fig. 7), the increases are more drastic, with more than 25% of the municipalities in Lebanon suffering from a 90% to more than 1 000% increase in domestic use. The percent increase in domestic use is crucial for highlighting stress on water infrastructure in the affected governorate or municipality. The absolute value of the increase in water use is a measure of the additional water quantity that needs to be secured to satisfy the incurring demand.

### Impact of the crisis on water stress

Assuming the Syrian crisis did not happen, the mean water stress in Lebanon would have been 0.5 at the end of 2016 (that is, the use is 50% of the total renewable surface and groundwater), which is above the high threshold of 0.4 but below the extremely high threshold of 0.8. Currently, and due to the refugee influx, mean water stress increased to 0.53 (6%), but it can be reduced from 0.53 to 0.49 if rehabilitation and water conservation measures are taken to improve efficiencies from 50 to 65%.

### Water stress at the watershed level

At the watershed level, watersheds in Lebanon show a crisis-induced increase in water stress of 2–10%. Out of 6.56 million, 2.45 are currently under extremely high water stress (compared to 2.1 million without the crisis), 3.2 million inhabitants live under high water stress (compared to 2.5 million without the crisis). Network improvements can reduce the population living under extremely high and high water stress by 10% (Table 2).

**Table 2:** Population living under low (less than 0.2), medium (between 0.2 and 0.4), high (between 0.4 and 0.8), and extremely high (greater than 0.8) water stress in Lebanon by watershed

Watershed	Population (excluding refugees) (scenario 1) (1 000 s)	Population (including refugees) (scenario 2) (1 000 s)	Water stress (scenario 1)	Water stress (scenario 2)	Water stress (scenario 3)
Beirut	1 524	1 690	1.68	1.81	1.42
Oustuene	135	180	1.29	1.35	1.29
Kabir	114	177	0.97	1.02	0.99
Rasain	372	408	0.87	0.89	0.83
Litani-North	420	803	0.64	0.70	0.66
Zahrani-Sinic	439	496	0.59	0.62	0.54
Barid-Arka	174	223	0.56	0.61	0.56
AboAli	682	818	0.55	0.61	0.52
Litani-South	327	373	0.52	0.54	0.51
Kalb	239	259	0.44	0.45	0.38
Damour	142	185	0.40	0.43	0.39
Jouz	107	122	0.42	0.43	0.40
Awali	138	180	0.29	0.32	0.28
Hasbani	110	133	0.22	0.23	0.22
Ibrahim	119	129	0.21	0.21	0.188
Orontes	237	389	0.19	0.20	0.194

Despite this increase, a potential for improvement exists in areas were network rehabilitation is possible. We find that, at the watershed level, decreasing network losses from 50 to 35% would lower water stress in many areas to better than pre-conflict levels (Table 2). At the municipal level, reducing losses from 50 to 35% will not be enough to reduce the stress to pre-crisis levels. Some 25% of the municipalities (mainly in the Bekaa and Akkar) need additional water sources to cope with the refugee crisis.

### Consequences of inaction

The consequences of inaction will eventually lead to decreased water supplies, deterioration in water quality, and a shift to relying more heavily on unsustainable groundwater pumping. These effects are already felt across the coastal zones through the massive saltwater intrusion in groundwater wells (El Moujabber et al. 2006; Masciopinto 2013), and through the decrease in groundwater levels in the Beqaa region (USAID 2011). Before the conflict and now, the Orontes part of the Beqaa has witnessed an increase higher than 20% in agricultural activity fed by groundwater (King and Jaafar 2015). The lax and unenforced governmental regulations for groundwater pumping will be an additional challenge. Climate change, although not factored herein, will create more stress. However, its impact in the short and medium-term is far less than the impact of the sudden increase in the refugee population in the country. The annual precipitation is expected to decline by around 2 mm/yr in the 21st century (Evans 2009).

Results of this work indicate that averaging water stress over the country would mask zones in the country that have much higher degrees of water stress than the national average. The higher the resolution of the stress analysis, the more hotspots will be uncovered. This is evident when comparing changes in domestic use at the governorate, the Kada, and the municipality level. For example, improving network efficiency at the country and governorate scale will bring domestic use to levels well below those induced by the refugee crisis. The same is not correct when running the analysis at the Kada or municipal level. The complexities of water network supplies necessitate demand–supply analysis at the level of network service, which is either the individual municipal level or the level of municipal conglomeration (i.e., an association of municipalities).

### Uncertainties and limitations

This study presents annual water stress estimates, and although the relative uncertainty in the comparative analysis between the pre-and post-crisis is low, the actual use estimates are affected by the per capita water-use figures. Population estimates are expected to be within ± 10% due to an estimated 250 000–350 000 tourists visiting the country over the year, mostly in summer. A negative net migration rate of − 0.6 per 1 000 for the 5 years before the crisis in 2011 is unlikely to affect the water-use estimates (World Bank 2016a). No internal migration data in Lebanon are collected (Bell et al. 2015). There are no actual measurements for industrial water use, and hence quantifying it as 30% of domestic use bears some uncertainty. There is also some degree of uncertainty in the CHIRPS precipitation estimates. While these biases in the precipitation estimates and the industrial use estimates will influence the magnitude of the freshwater stress index, they will not affect the results of the net increase in domestic water use. Also and due to lack of sufficient data, water transfers between governorates or municipalities within Lebanon were not included in the analysis.

### Recommendations

To alleviate water stress, there are three broad options—decrease population, increase the supply by increasing network efficiency, and reduce the use through environmentally sound technologies. Given the high number of Syrian refugees residing in Syria and no political solution in sight between the Assad regime and opposition forces, refugees are not likely to return en masse to Syria soon. While some may return voluntarily within the next year, that number is unlikely to be high enough that the population would reduce drastically enough in Lebanon to alleviate water stress. Further, because the Lebanese government has taken a nearly hands-off approach to the refugee crisis, it is not likely that they intend to play any role in reorganizing refugees within Lebanon, and the refugees themselves are unlikely to move from their current settlements to other parts of Lebanon. However, the government and the water authorities can work to implement several measures to conserve water at the national and regional scale. The refugee distribution within the country will probably remain static, barring some natural disaster or eruption of a conflict that forces them from their current settled locations.

Additionally, because urban water stress is already high, refugees should not be moved from rural to urban areas as it would serve to further exacerbate water stress in urban areas. Refugees in the Beqaa Valley have settled mainly on farmland. This causes its problems, namely, contamination of potable and irrigation water. Moving refugees from rural to urban areas would serve the same end as moving them from urban to rural areas and only worsen water stress in the Beqaa Valley. In either scenario, water stress levels would be further exacerbated by another increase in population. Because of the static refugee population, water stress must be addressed from a supply/water efficiency approach. Efforts must be undertaken to reshape the way water is thought about in Lebanon. Public awareness campaigns should be designed that frame water not as a political issue, but as a public good to which all citizens deserve access. These campaigns could also lead citizens to limit their consumption. Beyond efforts to reshape perceptions, other measures should be taken in the short term, such as introducing legislation that would implement metering and pricing schemes for domestic water consumption. While smart meters have begun to be installed in areas of Beirut, these efforts need to be more widespread throughout the country.

### Global implications

This research has global implications. As long as there is war, there will be refugee flows into neighboring host countries that will adversely affect the host country’s resources. It is important to note that refugees do not only cause undue harm on a host community, but they have been found to have positive effects as well, even in Lebanon. However, the depletion of natural resources, such as water, is a reasonable cause for concern in host communities, and more must be done to combat these effects. Sufficient water supplies must be made available early on in a crisis to deter illegal drilling of wells, such as connecting settlements to existing water networks. Care should also be taken to avoid establishing settlements in water-stressed areas. This requires more considerable planning and coordination between the international aid agencies and host governments early on in any crisis.

Given the scarcity of water that already exists in the region, it is vital to introduce mechanisms that will identify problem areas so that adverse effects of rising water stress caused by an influx in refugees may be diminished. The methodology used in this paper offers a framework for future analysis that can be used in other countries in the region and beyond to target hotspots of water stress. This will allow for further planning and the ability to introduce geographic-specific solutions to water stress in areas where there is a large population of refugees, such as in Turkey and Jordan. This will also be useful for emerging crises in the region. The region is volatile, and it is foreseeable that conflict may break out elsewhere in the region. Further, this same kind of analysis can be used outside the region in other places in the world that also host a large number of refugees. While some countries are more capable of coping with large refugee populations than others, the methodology can still be applied to help address potential problem areas.

## Conclusion

Water stress is an urgent issue in the Middle East, and rapid changes in population due to crisis put further strain on freshwater resources. This work was conducted to present an original estimate of the spatial water use at the national level in Lebanon. A water-use GIS-based analysis was performed by benchmarking, at several administrative and geography levels, the current water use (including agriculture) for all population in Lebanon against a no-refugee scenario based on Lebanese population estimated at the end of 2016. Results of our spatial analysis show that while the impact of refugees and indirectly conflicts’ on water stress is of paramount importance and it cannot be neglected, opportunities exist for the international community to intervene and provide for water supply and network efficiency improvements, which can relieve the induced stress.

Water stress transcends administrative boundaries, and it has increased today across the semi-arid region of Beqaa, as well as across many populated cities along the coastal zones of North and South Lebanon due to the refugee crisis. The recently induced increase in urban water stress requires increased political attention and resources. The refugee crisis question is politically sensitive, and a solution remains unknown. The existing situation, if left untreated, means that future urban water-use vulnerabilities will only worsen. Besides urban areas, rural areas would require special care in water network construction and rehabilitation efforts to meet the increasing uses. The resilience of rural livelihoods in Lebanon is highly dependent on sustained agricultural production areas where refugees are concentrated. If a political settlement is reached in Syria and refugees begin to return to Syria, several initiatives must be implemented to reduce water stress in Lebanon. Even as Syrian refugees return home and Lebanon’s population shrinks, water stress in Lebanon will remain a problem that must be addressed. These long-term initiatives include agriculture water-use reform and ultimately, government reform in the water sector.

## Electronic supplementary material

Below is the link to the electronic supplementary material.

Supplementary material 1 (PDF 1015 kb)

## References

[CR1] Abu-allaban M, El-Naqa A, Jaber M, Hammouri N (2015). Water scarcity impact of climate change in semi-arid regions: A case study in Mujib basin, Jordan. Arabian Journal of Geosciences.

[CR2] Achiume ET (2015). Syria, cost-sharing, and the responsibility to protect refugees. Minnesota Law Review.

[CR3] Bastiaanssen W, Noordman E, Pelgrum H, Davids G, Thoreson B, Allen R (2005). SEBAL model with remotely sensed data to improve water-resources management under actual field conditions. Journal of Irrigation and Drainage Engineering.

[CR4] Bastiaanssen WG, Menenti M, Feddes R, Holtslag A (1998). A remote sensing surface energy balance algorithm for land (SEBAL). 1. Formulation. Journal of Hydrology.

[CR5] Bell M, Charles-Edwards E, Kupiszewska D, Kupiszewski M, Stillwell J, Zhu Y (2015). Internal migration data around the world: Assessing contemporary practice. Population, Space and Place.

[CR6] Berti B (2015). The Syrian refugee crisis: Regional and human security implications. Strategic Assessment.

[CR7] Black R (1994). Forced migration and environmental change: The impact of refugees on host environments. Journal of Environmental Management.

[CR8] Bou-zeid E, El-fadel M (2002). Climate change and water resources in Lebanon and the Middle East. Journal of Water Resources Planning and Management.

[CR9] Central Administration of Statistics. 2017. *Population statistics*. Beirut: Central Administration of Statistics. http://www.cas.gov.lb/index.php/demographic-and-social-en/population-en. Accessed 15 Sept 2017.

[CR10] Dakroub, H. 2017. *Aoun appeals to world leaders on Syria refugee crisis*. Beirut, Lebanon: Daily Star. http://www.dailystar.com.lb/News/Lebanon-News/2017/Oct-17/422940-aoun-appeals-to-world-leaders-on-syria-refugee-crisis.ashx. Accessed 10 Nov 2017.

[CR11] Desa U (2014). World urbanization prospects, the 2011 revision.

[CR13] El-Khatib Z, Scales D, Vearey J, Forsberg BC (2013). Syrian refugees, between rocky crisis in Syria and hard inaccessibility to healthcare services in Lebanon and Jordan. Conflict and Health.

[CR14] El-Moujabber M, Samra BB, Darwish T, Atallah T (2006). Comparison of different indicators for groundwater contamination by seawater intrusion on the Lebanese coast. Water Resources Management.

[CR15] Evans JP (2009). 21st century climate change in the Middle East. Climatic Change.

[CR52] Falkenmark M, Rockström J (2006). The new blue and green water paradigm: Breaking new ground for water resources planning and management. Journal of Water Resources Planning and Management.

[CR16] FAOSTAT (2017). FAOSTAT database.

[CR17] Funk C, Peterson P, Landsfeld M, Pedreros D, Verdin J, Shukla S, Husak G, Rowland J, Harrison L, Hoell A, Michaelsen J (2015). The climate hazards infrared precipitation with stations—A new environmental record for monitoring extremes. Scientific Data.

[CR18] Gassert F, Landis M, Luck M, Reig P, Shiao T (2013). Aqueduct global maps 2.0.

[CR19] Hijmans, R. 2016. *Raster: Geographic data analysis and modeling*. R Package Version, Vol 2, 5–8.

[CR20] Hoerz T (1995). Refugees and host environments: A review of current and related literature.

[CR21] IAMP. 2016. *Informal settlements of syrian refugees in Lebanon*. https://data.humdata.org/dataset/. Accessed 10 June 2017.

[CR22] Jaafar HH (2014). Maximizing hydropower production from reservoirs: The case study of markaba. Lebanese Science Journal.

[CR23] Jaafar HH, Zurayk R, King C, Ahmad F, Al-Outa R (2015). Impact of the Syrian conflict on irrigated agriculture in the Orontes Basin. International Journal of Water Resources Development.

[CR24] Jaafar H, King-okumu C, Haj-hassan M, Abdallah C, El-Korek N, Ahmad F (2016). Water resources within the Upper Orontes and Litani Basins: A balance, demand and supply analysis amid the Syrian refugees crisis.

[CR25] Jaafar HH, Ahmad FA (2019). Time series trends of Landsat-based ET using automated calibration in METRIC and SEBAL: The Bekaa Valley, Lebanon. Remote Sensing of Environment.

[CR26] Jacobsen K (1997). Refugees’ environmental impact: The effect of patterns of settlement. Journal of Refugee Studies.

[CR27] King C, Jaafar H (2015). Rapid assessment of the water–energy–food–climate nexus in six selected basins of North Africa and West Asia undergoing transitions and scarcity threats. International Journal of Water Resources Development.

[CR28] Korfali S, Jurdi M (2010). Deterioration of coastal water aquifers: Causes and impacts. European Water.

[CR29] Masciopinto C (2013). Management of aquifer recharge in Lebanon by removing seawater intrusion from coastal aquifers. Journal of Environmental Management.

[CR53] McCabe MF, Wood EF (2006). Scale influences on the remote estimation of evapotranspiration using multiple satellite sensors. Remote Sensing of Environment.

[CR30] MOEW (2010). National water sector strategy: Supply/demand forecasts.

[CR31] Müller MF, Yoon J, Gorelick SM, Avisse N, Tilmant A (2016). Impact of the Syrian refugee crisis on land use and transboundary freshwater resources. Proceedings of the National Academy of Sciences.

[CR32] Oliver MA, Webster R (1990). Kriging: A method of interpolation for geographical information systems. International Journal of Geographical Information Systems.

[CR33] Paskett CJ (1998). Refugees and land use: The need for change in a growing problem. Journal of Soil and Water Conservation.

[CR34] Refaat MM, Mohanna K (2013). Syrian refugees in Lebanon: Facts and solutions. The Lancet.

[CR35] Roberts R (2010). Palestinians in Lebanon: Refugees living with long-term displacement.

[CR36] Running S, Mu Q, Zhao M (2017). MOD16A2 MODIS/Terra net evapotranspiration 8-day L4 global 500 m SIN Grid V006. NASA EOSDIS Land Processes DAAC.

[CR37] Safi A, Rachid G, El-Fadel M, Doummar J, Abou najm M, Alameddine I (2018). Synergy of climate change and local pressures on saltwater intrusion in coastal urban areas: Effective adaptation for policy planning. Water International.

[CR50] Sassoon, J. 2008. *The Iraqi refugees: The new crisis in the Middle East*. Bloomsbury Publishing.

[CR38] STATISTICS CAO. 2008. *Water consumption maps*. http://www.cas.gov.lb/images/Maps/Water_Subscriptions.pdf. Accessed Sept 2018.

[CR40] UNDP (2014). Lebanon environmental assessment of the Syrian conflict & priority interventions.

[CR41] UNDP (2014). Lebanon environmental assessment of the Syrian conflict & priority interventions.

[CR42] UNDP (2015). Rapid assessment of the impact of Syrian refugee influx on the environment in Jordan.

[CR43] UNHCR (2011). UNHCR resettlement handbook.

[CR44] USAID (2011). Litani river basin management support program: Water balance report.

[CR45] Wall M, Otis Campbell M, Janbek D (2017). Syrian refugees and information precarity. New Media & Society.

[CR46] World Bank (2010). Arab development assistance.

[CR47] World Bank. 2016a. *World development indicators*. Washington, DC: World Bank. http://data.worldbank.org/data-catalog/world-development-indicators. Accessed 9 Nov 2016.

[CR48] World Bank (2016). World: Forced displacement—A developing world crisis.

[CR51] World Bank. 2017. *World Bank* (2017, 10 December). Urban Population. Retrieved from https://data.worldbank.org/indicator/SP.URB.TOTL.IN.ZS.

