# Supplementary material for: Refugees, water balance, and water stress: Lessons learned from Lebanon
Source: Ambio. 2019 Nov 2;49(6):1179–93. doi: 10.1007/s13280-019-01272-0 (PMC7128003; doi:10.1007/s13280-019-01272-0)
Supplement: Supplementary file 1 — Supplementary material 1 (PDF 1015 kb) [file 13280_2019_1272_MOESM1_ESM.pdf]

Ambio

Electronic Supplementary Material
This supplementary material has not been peer reviewed

Title**: Refugees, water balance, and water stress: Lessons learned from Lebanon**

Hadi Jaafar, Farah Ahmad, Lauren Holtmeier, Caroline King-Okumu

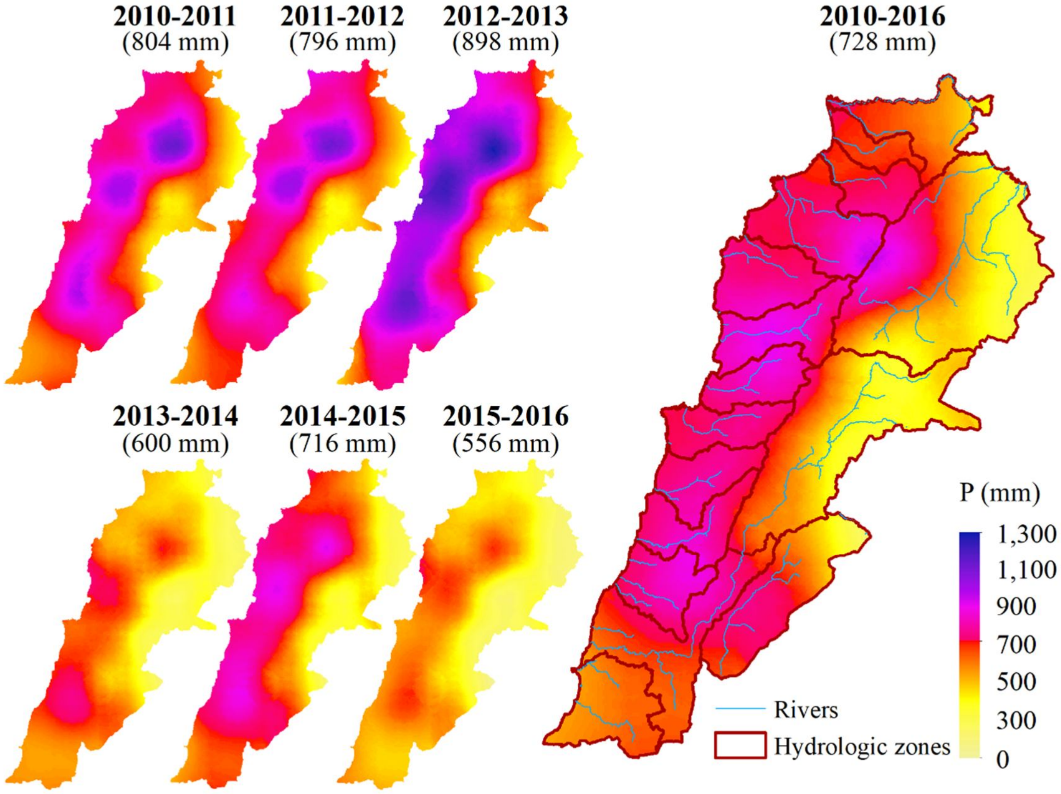

Figure S1. Variability in annual precipitation for Lebanon (2010-2016). Maps derived using a universal kriging interpolation of CHIRPS.

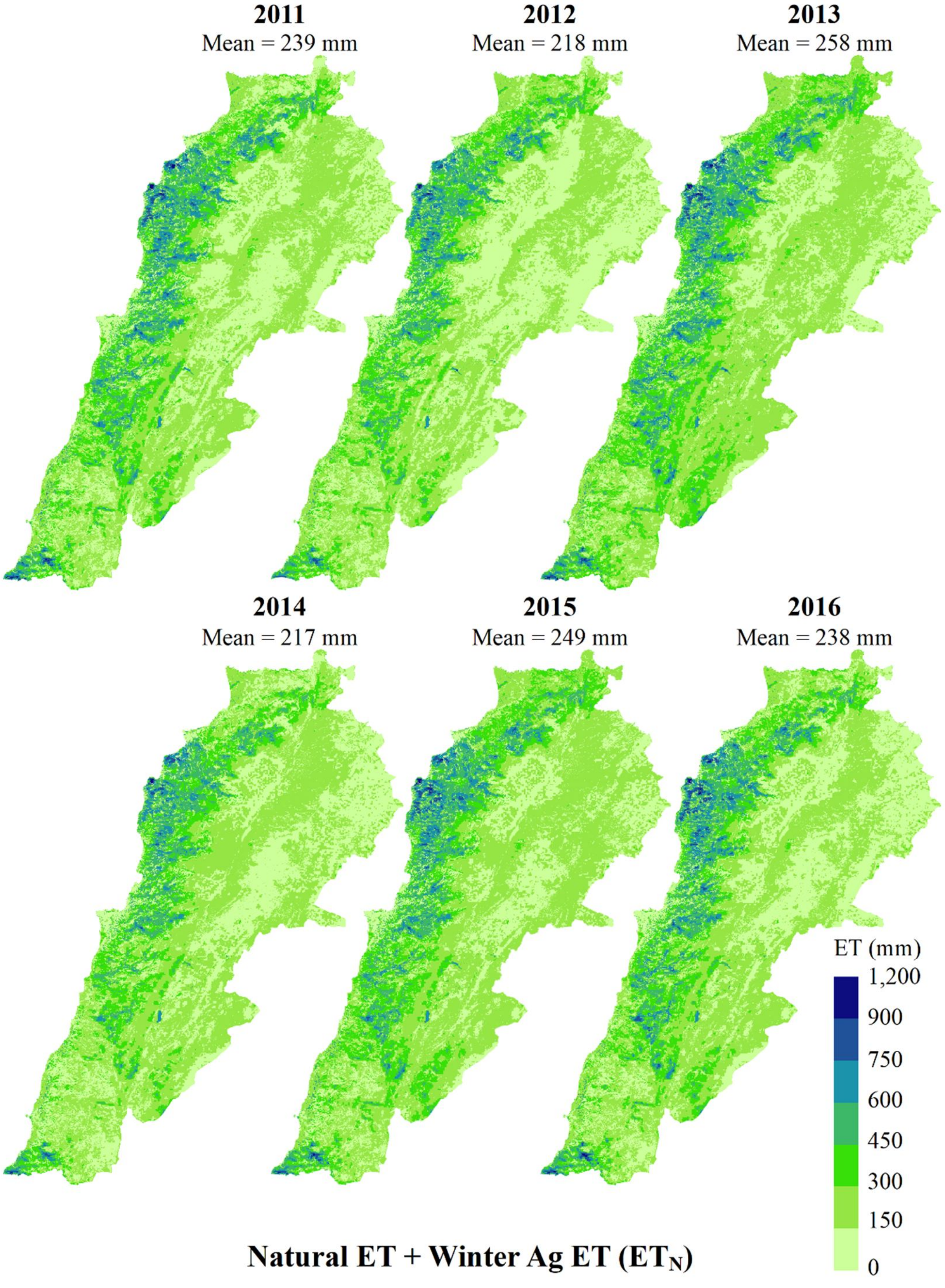

Figure S2. Variation of natural evapotranspiration and winter agricultural evapotranspiration in Lebanon

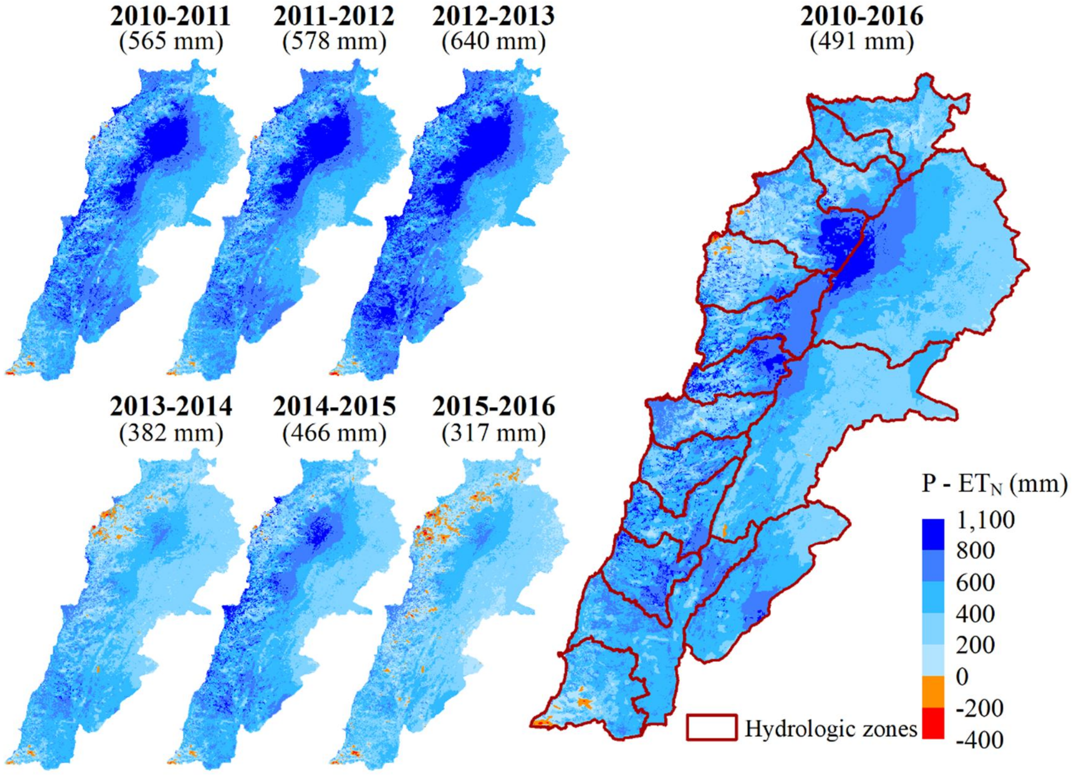

Figure S3. Variation of renewable water in Lebanon (2010-2016)
